# Clinical Protocols and Guidelines for Hypertension and Diabetes in Incarcerated Populations: A Scoping Review

**DOI:** 10.5888/pcd23.260139

**Published:** 2026-11-05

**Authors:** Raquel Alves de Oliveira, Davi Oliveira Teles, Samila Gomes Ribeiro, Paula Renata Amorim Lessa, Ana Izabel Oliveira Nicolau, Sabrina Freitas Nunes, Karine Nascimento da Silva, Camila Biazus Dalcin, Ana Karina Bezerra Pinheiro

**Affiliations:** 1 Department of Nursing, Federal University of Ceará, Fortaleza, Ceará, Brazil; 2 School of Health Sciences, University of Dundee, Nethergate, Dundee, United Kingdom

## Abstract

**Introduction:**

Incarcerated populations have a disproportionate burden of acute and chronic conditions, including hypertension and diabetes. We aimed to identify and describe clinical protocols and guidelines for managing hypertension and diabetes in the incarcerated population.

**Methods:**

We conducted a scoping review in July 2025 of clinical protocols and guidelines that address the prevention, promotion, screening, diagnosis, and treatment of hypertension and diabetes in the incarcerated population. We searched electronic databases (Medline/PubMed, Embase, the Virtual Health Library, EBSCOhost, Scopus, Cochrane, and Web of Science), gray literature repositories, and websites of governmental and international organizations. We used the AGREE II instrument to assess document quality.

**Results:**

Three documents met the inclusion criteria; they were published in 2021, 2022, and 2024 and originated from high-income countries. Methodological appraisal showed heterogeneity in guideline development, with consistently low scores in the domains of stakeholder involvement and rigor of development. Existing protocols encompass health education and self-management support, multidisciplinary care, universal screening at intake, evidence-based and individualized treatment, and intersectoral collaboration to ensure health care delivery in correctional settings.

**Conclusion:**

The small number of clinical protocols addressing hypertension and diabetes management in the incarcerated population reflects a critical normative gap in correctional health systems. Strengthening the scientific rigor of these protocols and ensuring their implementation are essential to standardize care, reduce clinical variation, and promote health equity, both within correctional settings and in the transition back to the community.

**Summary**

**What is already known on this topic?**

Incarcerated populations have a disproportionate burden of acute and chronic conditions. Structural constraints and health system fragmentation may compromise continuity and quality of care in correctional settings.

**What is added by this report?**

In our global scoping review, we identified only 3 prison-adapted clinical protocols for hypertension and diabetes, showing a critical normative gap in evidence-based guidance for chronic disease care in correctional health systems.

**What are the implications for public health practice?**

Methodologically rigorous, prison-adapted clinical protocols are needed to promote health equity in correctional settings and in the transition of incarcerated people back to the community.

## Introduction

Noncommunicable diseases account for approximately 75% of global deaths (1). Hypertension and diabetes are among the most prevalent conditions, affecting more than 1.28 billion (2) and 537 million adults worldwide, respectively, with diabetes projected to reach 783 million cases by 2045 (2,3). These trends highlight the need for effective prevention and management strategies.

The prison population is considered an inclusion health population, a term used for socially excluded groups who face overlapping social and health risk factors and heightened susceptibility to poor health outcomes (4). These vulnerabilities encompass individual (eg, biological, behavioral), social (eg, socioeconomic deprivation), and institutional (eg, health care access) dimensions. Inmates are disproportionately drawn from socioeconomically disadvantaged communities and have higher rates than the general population of mental illness, substance use, and communicable diseases, along with limited access to health care (4,5). Because prison environments influence continuity of care, health care costs, and community health after release, prison health is a public health issue (6).

Prison environments present structural barriers, including overcrowding, shortages of health care professionals, and disrupted continuity of care, that restrict health promotion and contribute to the development and worsening of noncommunicable diseases (5,7–9). Achieving equitable health outcomes requires additional efforts to overcome barriers related to prison organization and health care delivery (4,7).

Hypertension affects 36% of incarcerated men and up to 55% of incarcerated women (10), while diabetes affects approximately 14% of the global prison population (11,12). Although comparable to community estimates, these rates are notable because incarcerated populations are generally younger than the general population, suggesting earlier onset of cardiometabolic disease (13). Projections indicate that 6.4% of men in UK prisons — 33.3% among those aged 50 years or older — are at high risk of developing type 2 diabetes within the next decade (14). Associated complications include cardiovascular events, stroke, renal failure, foot ulceration, and amputation, the consequences of which may be severe in settings with limited resources (13,14).

The restrictive nature of prisons may compromise self-care and disease monitoring, while the lack of prison-adapted protocols contributes to fragmented and inequitable care (4,5,8,15). A review of management protocols for hypertension and diabetes is essential to provide support for adapted guidelines. These conditions are as relevant as infectious diseases, which receive greater attention in research on correctional health (10,13,14). Thus, synthesizing available evidence can support informed decision-making by professionals and managers. The objective of this review was to identify and describe clinical protocols and guidelines for the management of hypertension and diabetes in the incarcerated population.

## Methods

### Study design

We conducted this scoping review following the methodological framework proposed by the Joanna Briggs Institute (16) and reported in accordance with the Preferred Reporting Items for Systematic Reviews and Meta-Analyses Extension for Scoping Reviews (PRISMA-ScR) (17). 

### Research question

We structured the research question according to the PCC (Population, Concept, Concept) framework recommended by the Joanna Briggs Institute, in which Population (P) was defined as incarcerated adults; Concept (C) was defined as clinical protocols and guidelines addressing the promotion, prevention, screening, diagnosis, and treatment of hypertension and/or diabetes; and Context (C) was defined as prison health systems, including correctional facilities, penitentiaries, and detention establishments across any level of health care delivery in any country. Accordingly, the following research question guided the investigation: What are the clinical protocols and guidelines in the scientific literature on the promotion, prevention, diagnosis, screening, and treatment of hypertension and diabetes in prison populations?

### Databases and search strategy

We used the following electronic databases to conduct the search in July 2025: Medical Literature Analysis and Retrieval System Online/National Library of Medicine (Medline/PubMed), the Virtual Health Library (which includes several Latin databases), Embase, EBSCOhost, Scopus, Cochrane Protocols, and Web of Science.

For gray literature, we used the following databases: ProQuest, the Brazilian Digital Library of Theses and Dissertations, and Banco de Dados em Enfermagem, a bibliographic database specialized in nursing. We conducted searches on the Coordination for the Improvement of Higher Education Personnel portal via institutional remote access to the Federal University of Ceará. In addition, we searched repositories of clinical protocols, guidelines, and websites of governmental organizations: the National Institute for Health and Clinical Excellence (United Kingdom); the National Health and Medical Research Council (Australia); the Canadian Medical Association InfoBase (Canada); the Guidelines International Network (Scotland); the World Health Organization (WHO [Switzerland]); the Pan American Health Organization (PAHO [US]); the Brazilian Ministry of Health (Brazil); the National Council of Justice (Brazil); the National Commission for the Incorporation of Technologies in the Unified Health System (Brazil); and CorrectCare, the magazine of the National Commission on Correctional Health Care (US).

The search strategy was developed using controlled descriptors from Medical Subject Headings (MeSH) for most databases, whereas for Embase, search terms included the corresponding Emtree preferred terms adapted with tree explosion (/exp). The overall strategy combined terms related to guidelines or protocols, incarcerated populations, and chronic conditions: (“Guideline” OR “Practice Guideline” OR “Clinical Guidelines” OR “Clinical Practice Guideline” OR “Clinical Protocols” OR “Clinical Protocol” OR “Protocol, Clinical” OR “Protocols, Clinical” OR “Protocols, Treatment” OR “Treatment Protocol” OR “Treatment Protocols”) AND (Prisoners OR Prisoner OR Prisons OR Penitentiaries OR Penitentiary OR Prison) AND (“Hypertension” OR “Diabetes Mellitus”). Search strategies were tested and adapted for each database to maximize sensitivity. In the gray literature search, where advanced search features were often unavailable, isolated terms or combinations (including “Clinical Protocols,” “Guideline,” “Hypertension,” and “Diabetes Mellitus”) were applied, using publication guides to direct the search. Screening across databases and repositories involved tailored strategies to identify all available protocols (Appendix).

### Eligibility criteria

Inclusion criteria targeted protocols and clinical guidelines for hypertension and/or diabetes management that encompassed prevention, promotion, screening, diagnosis, and treatment in the prison system. We selected only documents entirely focused on care for the incarcerated population, with no restrictions on publication date, language, or population sex. Exclusion criteria included documents lacking scientific substantiation of recommendations or electronic full-text availability.

### Screening and selection process

We exported all identified studies to Rayyan software (Rayyan Systems, Inc) for duplicate removal. Two independent researchers (R.A.O. and D.O.T.) conducted a 2-stage screening process: an initial title and abstract review followed by full-text evaluation of eligible documents. Discrepancies were resolved by a third researcher (S.G.R.) after unblinding. For gray literature incompatible with Rayyan, we managed files via Google Drive for independent review and consensus-based selection.

### Data charting

Two trained reviewers (R.A.O. and D.O.T.) independently charted data using a standardized Google Sheets form. The charted variables included responsible organization and authors; title; publication date; country and language; target population profile; type of prison; involved health professionals; and clinical recommendations for hypertension and diabetes prevention, promotion, screening, diagnosis, and treatment.

### Quality appraisal

Although formal quality appraisal is optional in scoping reviews (18), we applied the Appraisal of Guidelines Research and Evaluation (AGREE II) instrument (18,19) to evaluate the methodological rigor of the included documents and identify those that offer structured guidance for practitioners and policymakers. Two independent reviewers (R.A.O. and D.O.T.) applied the instrument, which features 23 items organized into 6 domains plus 2 global rating items, each scored on a 7-point Likert scale (from 1, strongly disagree, to 7, strongly agree). Domain scores were standardized as percentages of the maximum possible score, with higher scores indicating higher methodological quality (18,19).

The 6 domains are scope and purpose (3 items), stakeholder involvement (3 items), rigor of development (8 items), clarity of presentation (3 items), applicability (4 items), and editorial independence (2 items) (19). Standardized domain scores for each document were calculated as percentages: (obtained score − minimum possible score) divided by (maximum possible score − minimum possible score). The global rating was calculated for each document as the mean of the 2 global items (overall quality and recommendation for use) across 2 reviewers (R.A.O. and D.O.T.).

### Synthesis of evidence

We grouped recommendations by levels of health care continuum (prevention, promotion, screening, diagnosis, and treatment) and compared them across included documents to identify areas of convergence and divergence. We synthesized document characteristics and AGREE II results in tables and constructed a recommendation matrix to facilitate visual comparison of similarities and discrepancies across documents.

## Results

The initial search yielded 2,682 records from electronic databases and 1,969 from gray literature sources (Figure 1). After removing 250 duplicates and screening titles and abstracts, we selected 383 database records and 60 gray literature documents for full-text review. After independent full-text evaluation and consensus resolution, 2 documents met all predefined inclusion criteria. A third document, the American Diabetes Association (ADA) Statement on Diabetes Management in Detention Facilities (20), was identified during the peer review process at *Preventing Chronic Disease* by a peer reviewer and was assessed against the inclusion criteria by 2 independent reviewers (R.A.O. and D.O.T.). The document met all eligibility requirements and was formally incorporated into our analysis. Ultimately, we included 3 documents in this review.

**Figure 1:**
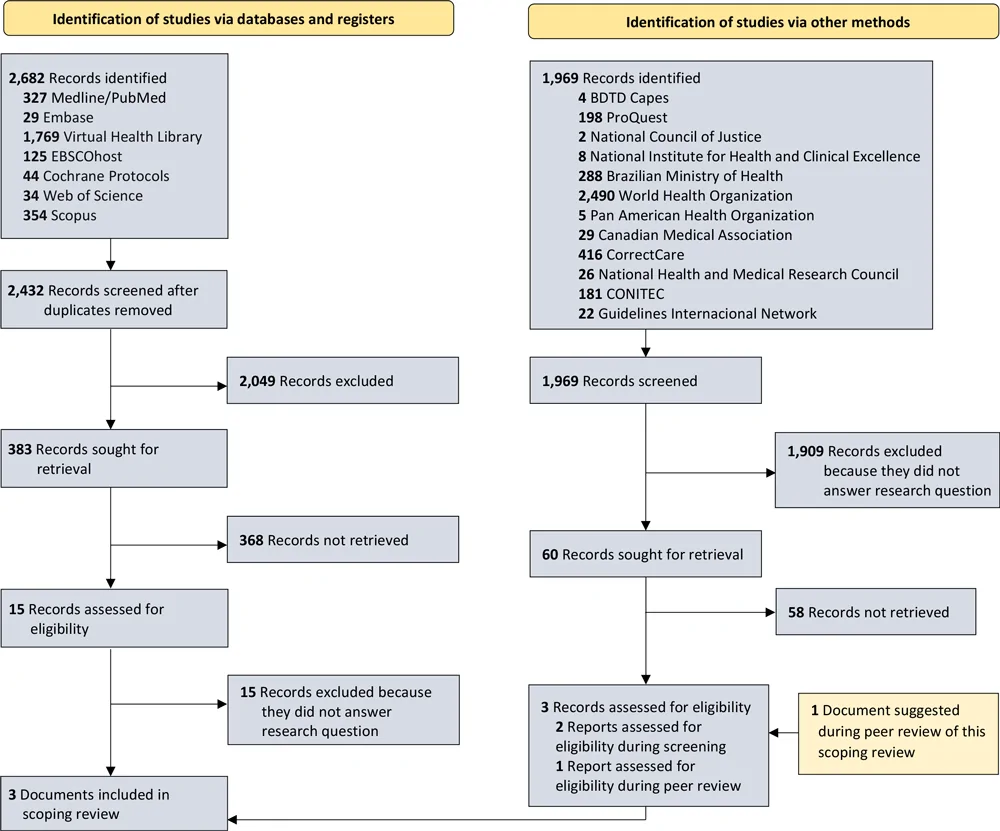
Flowchart of the study selection process for a scoping review of clinical protocols and guidelines for hypertension and diabetes for incarcerated populations. Adapted from the PRISMA-ScR flowchart (17). Abbreviations: BDTD, Brazilian Digital Library of Theses and Dissertations; CONITEC, The National Commission for the Incorporation of Technologies in the Unified Health System.

The 3 included documents were published in 2021, 2022, and 2024 and originated from high-income countries: 1 from Denmark (21), and 2 from the US (20,22) (Table 1). All were published in English, focused exclusively on custodial environments, and involved multidisciplinary health care teams in the delivery of recommendations (Table 2). The ADA statement is the most clinically detailed of the 3 documents, providing operational guidance across the full continuum of care, from intake screening within the first 2 hours of arrival to discharge planning beginning at least 1 month before release. The WHO Prison Health Framework is a system performance assessment tool, organizing prison health through building blocks (health system inputs, service delivery outputs, and health outcomes). The Federal Bureau of Prisons (FBOP) document focuses on preventive health care screening in the federal prison system. 

**Table 1:** Characteristics of the Documents Included in a Scoping Review of Clinical Protocols and Guidelines for Hypertension and Diabetes for Incarcerated Populations

Document	Country	Title	Context of application	Professionals involved
World Health Organization (21), 2021	Denmark	The WHO prison health framework: a framework for assessment of prison health system performance	Recommendations for the prison system	Multidisciplinary team/prison system
Federal Bureau of Prisons (22), 2022	US	Preventive health care screening: Federal Bureau of Prisons clinical guidance	Recommendations for the prison system (federal prison)	Multidisciplinary team/prison system
American Diabetes Association (20), 2024	US	Diabetes management in detention facilities: a statement of the American Diabetes Association	Recommendations for detention facilities	Multidisciplinary team/detention facilities

**Table 2:** Recommendations Found in Clinical Protocols and Guidelines Documents for Health Promotion, Prevention, Screening, Diagnosis, and Treatment of Hypertension and Diabetes for Incarcerated Populations

Level of health care continuum	Convergent recommendations (present in ≥2 documents)	Exclusive or additional recommendations		
		World Health Organization (21), 2021	Federal Bureau of Prisons (22), 2022	American Diabetes Association (20), 2024
Promotion	**Health education and staff training** Educational campaigns on healthy eating, physical activity, smoking, and alcohol cessation Continuous training of prison health care teams and ethical practice in care delivery **Patient engagement** Encouraging incarcerated individuals to seek health services; guiding shared responsibility for health Tobacco, drug, and alcohol cessation support	Existence and implementation of smoke-free prison policies as a measurable performance indicator Rehabilitation programs (educational, employment training) tracked as part of health service delivery Inmate health and well-being (self-reported) as a health outcomes indicator	Educational DVDs and health fairs conducted by volunteers and community organizations Inmate patient fact sheets (sex-specific) covering all preventive health topics	**Structured diabetes self-management education program (at least biannually)** Hypoglycemia/hyperglycemia recognition, medications, monitoring, foot care, meal planning, psychosocial adjustment **Formal custodial staff education curriculum** Signs/symptoms of diabetes, emergency response to hypoglycemia/hyperglycemia, glucose monitoring, nutrition and meal timing Behavioral strategies: motivational interviewing, goal setting, problem-solving, facilitation of social support Integration of psychosocial/mental health assessment into routine diabetes care Cannabis-associated diabetic ketoacidosis risk awareness
Prevention	**Nutrition and physical activity** Ensure adequate nutrition and physical activity in the prison environment **Cardiovascular pharmacological prevention** Aspirin therapy and statin therapy for CVD risk reduction in eligible adults Substance use counseling and cessation support	Harm reduction services: availability of needle/syringe programs, condoms Nutritional options aligned with cultural and gender needs as a system performance indicator Smoke-free prison policies as a health protection indicator	Nutrition/exercise counselling and referral to weight reduction clinic for body mass index ≥30 kg/m^2^ 10-year CVD/stroke risk calculation every 5 years (American College of Cardiology/American Heart Association calculator) Folic acid supplements (400–800 µg) for women of childbearing age	**Nutritionally balanced menus** Consistent carbohydrate content at each meal; macronutrient content disclosed per meal selection Snacks with fast-acting carbohydrates for individuals using insulin; commissary staff trained in hypoglycemia treatment Meal/snack timing coordinated with medication schedules; glucose testing and insulin dosing within 15 minutes before meals Preference for low-hypoglycemia–risk medications (biguanides, DPP-4i, GLP-1 RA, SGLT2i) given limited monitoring access Diabetes patients with complex detoxification needs referred to equipped facility
Screening	**Universal intake assessment** All incarcerated individuals assessed at entry for chronic diseases, mental health, and substance use **Blood pressure screening** All adults aged ≥18 years; more frequent for those aged ≥40 years or with risk factors **Diabetes screening** Fasting glucose or HbA_1c_; all aged ≥45 years and overweight/obese aged 35–70 years; reassess every 3 years if negative Cardiovascular risk stratification at intake and follow-up visits	Population-level noncommunicable disease morbidity tracking (hypertension, CVD, diabetes, cancer) as system performance indicators Infectious disease screening indicators (tuberculosis, HIV, hepatitis C virus, hepatitis B virus, sexually transmitted infections) at admission	Cognitive impairment screening (Mini-Cog) for those aged ≥50 years Osteoporosis: bone mineral density screening (DXA scan) for women aged ≥65 years and risk-based for younger women Lipid profile annually for those aged ≥20 years with diabetes/CVD; every 5 years for average risk for those aged ≥40 years Obesity: height, weight, and body mass index calculation at every preventive visit	**Reception screening (within 1–2 hours)** Identify insulin users; blood glucose monitoring and urine ketone test; continue usual medications and meals. Any altered mental status or diaphoresis should lead to immediate glucose check (hypoglycemia may mimic intoxication) **Intake screening (within 2–24 hours)** Diabetes type and duration; current therapy; complications; pregnancy; alcohol or drug use; behavioral health (depression, suicidal ideation); prior diabetes education **Physical examination plus laboratory (within 2 hours to 2 weeks)** Retinal examination, cardiac examination, peripheral pulses, foot/neurologic examination; HbA_1c_, lipids, microalbumin, creatinine/eGFR **Ongoing blood glucose monitoring** Type 1: ≥3 times per day or continuous monitoring. Type 2 and using insulin: ≥1 times per day. HbA_1c_: ≥2 times per year if at goal, quarterly if not **Screening for complications** Annual retinal examination, urinary albumin + eGFR; ankle-brachial index (peripheral arterial disease) for those aged ≥50 years with microvascular disease or with diabetes for ≥10 years
Diagnosis	**Comprehensive history and physical examination** Medical history, risk factors, anthropometric measurements, target organ damage assessment **Multidisciplinary team** All health care staff involved; unrestricted access to incarcerated individuals **Electronic health records:** Risk assessment forms; individualized follow-up plan; data integrated where possible in national health system Fasting glucose or HbA_1c_ for diabetes diagnosis; blood pressure measurement for hypertension diagnosis	Health information system indicators: existence of health records, exhaustiveness, integration in national system, death-in-custody recording Standardized procedure for identifying people at risk of suicide/self-harm Assessments of availability of essential medicines (World Health Organization’s Essential Medicines List)	Federal Bureau of Prisons electronic health record forms for preventive risk assessment at baseline visit 10-year CVD risk calculation; aspirin/statin therapy triggered by calculated risk	**Identification of** **diabetes type at intake** Any individual using insulin presumed type 1 until confirmed otherwise; type 1 requires more intensive monitoring **Technology continuity at admission** Uninterrupted access to continuous glucose monitor, insulin pumps, and automated delivery systems unless individualized security risk identified **Psychosocial/mental health diagnosis** Incorporated into routine care; clinically significant mental health diagnoses more prevalent in people with diabetes **Individualized glycemic targets** HbA_1c_ ≤7% for most; less stringent for history of severe hypoglycemia, limited life expectancy, elderly, certain comorbidities Thyroid disease screening for type 1 diabetes at diagnosis and periodically thereafter
Treatment	**Evidence-based, individualized management** Lifestyle modification as first line; pharmacotherapy accessible and tailored to comorbidities **Universal access to medications** Antihypertensive and diabetes medications provided; health care costs covered by the state; no copayments or fees **Multidisciplinary, longitudinal follow-up** **Continuity of care** Medication reconciliation at admission; supply of medications upon release **Cardiovascular risk pharmacotherapy** Aspirin and statin therapy for eligible patients	Standardized procedures for reporting medication errors and adverse drug reactions Community linkage: registration with a general practitioner after release Equivalence principle: same scope and quality of care as available in the community	Aspirin: low-dose (75–100 mg/d) for atherosclerotic CVD primary prevention for those aged 40–70 years; not recommended for those aged >70 years or if at increased risk of bleeding Statin: moderate-intensity for CVD risk ≥7.5% for those aged 40–75 years	**Type 1 diabetes** Basal and prandial insulin regimen; sliding-scale insulin alone strongly discouraged; insulin pump/continuous glucose monitor considered when feasible **Type 2 diabetes** Prefer low-hypoglycemia–risk agents; proactive standing-dose adjustment; avoid sole sliding scale **Emergency protocols** Hypoglycemia (15–20 g carbohydrates; glucagon available); diabetic ketoacidosis (ketone testing, urgent evaluation; euglycemic diabetic ketoacidosis with SGLT2i) **Transfer** Medical summary with diagnosis, medications, last dose, recent results, risk flags; supplies accompany individual **Discharge planning** Begins ≥1 month before release: community resources, follow-up appointment, medication supply, written health summary Telehealth for diabetes specialist consultation when on-site expertise unavailable (14) Special populations: pediatric and pregnancy-specific protocols

Abbreviations: CVD, cardiovascular disease; DPP-4i, dipeptidyl peptidase-4 inhibitor; DXA, dual-energy x-ray absorptiometry; eGFR, estimated glomerular filtration rate; GLP-1 RA, glucagon-like peptide-1 receptor agonist; HbA_1c_, glycated hemoglobin A_1c_; SGLT2i, sodium-glucose cotransporter-2 inhibitor.

### Health promotion

All 3 documents converge on the importance of health education, staff training, and encouraging incarcerated people to take active responsibility for their health. The WHO framework and the FBOP document both address educational campaigns on healthy eating, physical activity, and substance use, and the FBOP document provides concrete delivery formats such as fact sheets, health fairs, and audiovisual resources. The ADA statement converges on these themes but adds a qualitatively distinct layer: a structured diabetes self-management education program with defined curricular components, and a formal custody staff training curriculum to be delivered at least biannually, elements absent from the other 2 documents. Psychosocial integration, behavioral activation strategies, and awareness of the risk of cannabis-associated diabetic ketoacidosis are also exclusive to the ADA statement.

### Prevention

Adequate nutrition, physical activity, and cardiovascular pharmacological prevention (aspirin and statin therapy) were the main areas of convergence across the 3 documents. The WHO framework addresses prevention through system-level indicators (harm reduction services, smoke-free policies, nutritional adequacy) rather than clinical prescriptions. The ADA statement and FBOP document specify aspirin and statin thresholds for atherosclerotic cardiovascular disease prevention, although with slightly different age cutoffs. The ADA statement diverges considerably on nutritional prevention, specifying consistent carbohydrate content at each meal, snack provision for insulin users, meal-timing coordination with medications, and a preference for low-hypoglycemia–risk pharmacological agents; these operational recommendations have no equivalent in the other 2 documents.

### Screening

Discussion of universal intake assessment and regular blood pressure and diabetes screening converge across the 3 documents. The ADA statement and the FBOP document are aligned on specific screening intervals: blood pressure screening for those aged 18 years or older (more frequent for those aged ≥40 y or those with risk factors); diabetes screening with fasting glucose or glycated hemoglobin A_1c_ (HbA_1c_) for those aged 45 years or older and for those aged 35 to 70 years with overweight or obesity, with retesting every 3 years if initial diabetes test results are negative. The WHO framework contributes prison population-level tracking of deaths due to noncommunicable diseases as system performance indicators rather than as individual screening protocols. The most pronounced divergence is in the 3-tiered timeline for intake screening in the ADA statement: insulin users should be identified, and glucose should be checked within 1 or 2 hours of intake; mental health as well as diabetes type, therapy, and complications should be assessed within 2 to 24 hours; and a full physical and laboratory evaluation should be conducted within 2 hours to 2 weeks, a level of operational specificity entirely absent from the other 2 documents. Frequency of ongoing glucose monitoring and intervals of surveillance for complications (retinopathy, nephropathy, peripheral arterial disease) are also exclusive to the ADA statement.

### Diagnosis

Comprehensive history-taking, physical examination, and anthropometric assessment at intake converge across the 3 documents, as do blood pressure measurement for hypertension and the use of fasting glucose or HbA_1c_ for diabetes diagnosis. The WHO framework and the FBOP document both emphasize multidisciplinary teams and integration of electronic health records. The WHO framework adds system-level diagnostic infrastructure indicators, health records, national system integration, identification procedures for suicide and self-harm, and medicine availability assessments. The FBOP document includes a structured 10-year cardiovascular risk calculation to trigger treatment decisions. The ADA statement diverges on several clinically critical elements: mandatory identification of diabetes type at intake (with type 1 presumed until confirmed); uninterrupted continuity of diabetes technology (continuous glucose monitor, insulin pumps); psychosocial and mental health diagnosis incorporated into routine care; and individualized glycemic targets, none of which are addressed by the other 2 documents.

### Treatment

The broadest area of convergence across all 3 documents is treatment: evidence-based individualized management, lifestyle modification as first-line therapy, universal access to medications, multidisciplinary longitudinal follow-up, and medication reconciliation at admission. The ADA statement and the WHO framework both explicitly affirm that health care costs must be covered by the state with no copayments charged to incarcerated individuals, a financing principle absent from the FBOP document. The ADA statement and the FBOP document converge on thresholds of cardiovascular pharmacotherapy (aspirin and statin criteria). The most extensive divergence is again found in the ADA statement: type-specific insulin regimens (strongly discouraging sole reliance on sliding-scale insulin); emergency protocols for hypoglycemia and diabetic ketoacidosis; medication transfer summaries; discharge planning beginning at least 1 month before release; telehealth for specialist consultation; and dedicated protocols for pediatric patients and pregnancy. These recommendations offer operational guidance for which no equivalent exists in the WHO framework or FBOP document, and they address diabetes management, reinforcing the asymmetry between hypertension and diabetes in available normative guidance.

Notably, none of the documents provides formal levels of evidence for their recommendations. 

### AGREE II domain scores

We found heterogeneous AGREE II domain scores across the 3 documents (Figure 2). “Stakeholder involvement” varied considerably, with the FBOP document showing particularly limited evidence of participation from key groups, including health care professionals and incarcerated individuals. “Rigor of development” received the lowest scores, indicating a shared weakness in methodological transparency. The “applicability” domain received low scores across all documents. “Editorial independence” received low scores in WHO and ADA documents, due to limited disclosure of funding sources and conflicts of interest, the FBOP document score in this domain was zeroed due to the complete lack of information regarding editorial independence. 

**Figure 2:**
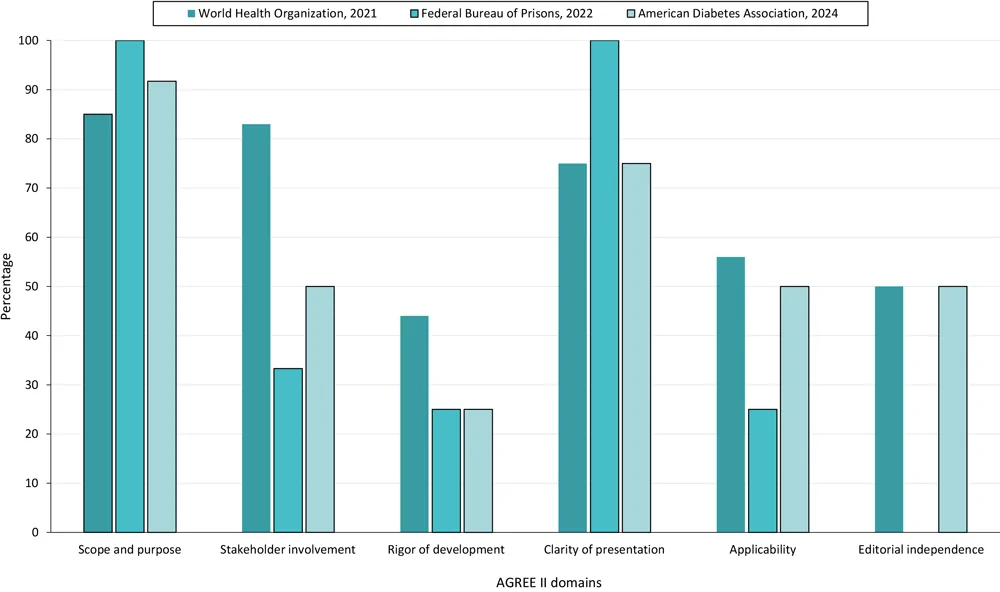
Scores for the adequacy of guidelines included in a scoping review of clinical protocols and guidelines for hypertension and diabetes for incarcerated populations according to AGREE II domains. Domain scores for each document were calculated as percentages: (obtained score − minimum possible score) divided by (maximum possible score − minimum possible score). The higher the score, the better the adequacy. Abbreviation: AGREE II, Appraisal of Guidelines for Research and Evaluation II.

**Table:** 

Document	Scope and purpose	Stakeholder involvement	Rigor of development	Clarity of presentation	Applicability	Editorial independence
World Health Organization, 2021	85	83	44	75	56	50
Federal Bureau of Prisons, 2022	100.0	33.3	25	100	25	0
American Diabetes Association, 2024	91.7	50	25	75	50	50

Conversely, “scope and purpose” and “clarity of presentation” received consistently high scores, reflecting well-defined objectives and clear organization. Mean global rating scores (1–7 scale) were 6.7 for the ADA statement (overall high quality), 6.5 for the WHO framework (overall high quality), and 4.5 for the FBOP document (moderate quality).

## Discussion

This scoping review identified only 3 documents addressing the management of hypertension and/or diabetes in the incarcerated population. Our ability to retrieve only 3 documents after an extensive search across 22 sources confirms that the normative gap in prison-adapted clinical protocols is not a bibliographic artifact but a structural absence, consistent with the documented neglect in correctional health research of noncommunicable diseases relative to communicable diseases (10,23).

All documents originated from high-income countries, reflecting a geographic concentration that must be acknowledged. Most of the world’s incarcerated population is held in low- and middle-income countries, where evidence related to correctional health is severely limited (24). The transferability of recommendations developed in high-income countries — such as continuous glucose monitoring, insulin pumps, and structured discharge planning — to resource-constrained settings cannot be assumed and represents a critical agenda for future research.

Structural barriers to prison health care must be acknowledged: overcrowding, shortages of health care professionals, staff turnover, and insufficient funding consistently undermine even delivery of basic care (22,25). Many recommendations in the included documents are aspirational in most prison systems, where structural conditions for their implementation do not exist (23). This gap between aspiration and actual delivery calls for intersectoral action on the systemic determinants of prison health care quality.

A concerning convergent finding is the low AGREE II score for “rigor of development” across the 3 documents, indicating shared weakness in methodological transparency. This systemic pattern is confirmed across medical specialties (26,27) and reproduced here in correctional health. Without methodological transparency, it is not possible to verify whether recommendations reflect the best available evidence or to update them as evidence evolves.

AGREE II scores for “stakeholder involvement” varied substantially. The WHO framework scored highest for this domain because WHO engaged a 27-institution steering group and 12 technical experts. The ADA statement held an intermediate position because ADA engaged with a specialized clinical group. The FBOP document scored lowest, reflecting FBOP’s reliance on internal governmental guidance that lacked documented input from external stakeholders, frontline staff, or incarcerated individuals. This shortfall aligns with literature reporting stakeholder involvement as a recurrent bottleneck in clinical guidelines, often hampered by institutional barriers to recruitment and definitions of stakeholder role (26,27). Critically, guidelines developed without diverse stakeholder representation risk failing to incorporate the social realities and operational values of patients (27). As evidence shows, poor stakeholder integration directly compromises implementation and practical use in complex settings (27).

Despite convergence on universal intake screening, multidisciplinary care, individualized management, lifestyle modification, and publicly funded health care, the documents differ substantially in operational depth. The ADA statement is by far the most operationally detailed, covering intake timelines, glycemic targets, insulin regimens, emergency protocols, transfer procedures, and discharge planning. The WHO framework functions as a governance and performance assessment instrument; the FBOP document offers screening guidance with limited details on treatment. This asymmetry reveals that diabetes has received more normative attention than hypertension, a disparity warranting prioritization in future development of guidelines.

A qualitative study in Thai prisons (25) provides an empirical counterpoint to these aspirational recommendations, documenting resource constraints, inequalities in screening, and self-reliance as the dominant realities of hypertension and diabetes management in custodial settings, an implementation gap relevant not only to low- and middle-income contexts but to underresourced facilities globally (23,25).

The intersection of chronic disease and demographic aging demands urgent attention. Older incarcerated individuals (those aged ≥50 y) constitute the fastest growing group in many prison systems (28). A systematic review, which reported a high prevalence of noncommunicable diseases in prison populations, focused on this age group (29); standard protocols were not designed to address their multimorbidity and polypharmacy (28). As prison populations continue to age, a trend accelerated by longer sentences and restricted access to early release in many jurisdictions, the normative gap in prison-adapted clinical protocols identified by this review will become progressively more consequential.

The growing global burden of hypertension and diabetes, combined with increasing and aging prison populations, makes the development of robust, evidence-based, context-adapted protocols an urgent public health priority, not only for correctional systems but for the broader communities to which incarcerated individuals will return. Prison health is inseparable from public health: continuity of care at release, medication supply, and connection to community health care services determine whether health gains made during incarceration are sustained or rapidly eroded (30,31).

This scoping review reveals a thematic gap between hypertension and diabetes. Although the ADA statement (20) provides comprehensive guidance on diabetes care in detention, no document with equivalent clinical operationalization exists for hypertension management. Future guideline development should prioritize hypertension as urgently as diabetes, given its high prevalence, reported at 36% in incarcerated men and 55% in incarcerated women (10,11), and its role as a leading cause of cardiovascular morbidity and mortality in this population (12).

### Strengths and limitations

This review is strengthened by its extensive multisource search strategy, covering 22 academic and gray literature sources and producing a comprehensive map of available normative guidance. The use of the AGREE II instrument for structured quality appraisal enabled comparison of methodological rigor.

Limitations include the geographic concentration in high-income countries of included documents, which restricts the generalizability of findings to other correctional contexts. The scarcity of documents also limited the depth of comparative analysis. Finally, clinical protocols are frequently internal institutional documents not indexed in academic databases, a structural characteristic of this documentary category that limits retrieval independent of search comprehensiveness.

### Conclusion

This scoping review identified 3 clinical protocols addressing the management of hypertension and diabetes in the incarcerated population, revealing a critical normative gap in correctional health systems. The evidence emphasizes that structured health education, self-care promotion, and multidisciplinary care are essential components of effective chronic disease management in custodial settings. Prevention strategies should prioritize nonpharmacological measures, including adequate nutrition and physical activity, while screening and diagnosis require universal risk stratification, systematic intake assessment, and regular monitoring adapted to the specific conditions of the prison environment. Treatment must be individualized, ensure universal access to medications without financial barriers, and position lifestyle modification alongside pharmacotherapy within a longitudinal, multidisciplinary care framework.

The scarcity of specific and methodologically rigorous clinical protocols and guidelines highlights an urgent need for the development of evidence-based clinical guidelines tailored to the prison environment. These guidelines should be applicable across diverse resource settings and developed with meaningful participation of health care professionals, managers, and the incarcerated population. Strengthening the scientific quality of these instruments is vital not only to guide and improve care within correctional facilities but also to support continuity of care upon release and promote health equity.

## Appendix Search Strings for Each Source in a Scoping Review of Clinical Protocols and Guidelines for Hypertension and Diabetes for Incarcerated Populations

**Table:**

Database	Search query 2025, Jul 26th
Virtual Health Library, which includes many Latin-American databases	(((((((((((Guideline) OR (Practice Guideline)) OR (Clinical Guidelines)) OR (Clinical Practice Guideline)) OR (Clinical Protocols)) OR (Clinical Protocol)) OR (Protocol, Clinical)) OR (Protocols, Clinical)) OR (Protocols, Treatment)) OR (Treatment Protocol)) OR (Treatment Protocols)) AND ((((((Prisoners) OR (Prisoner)) OR (Prisons)) OR (Penitentiaries)) OR (Penitentiary)) OR (Prison))
Scopus	(TITLE-ABS-KEY (guideline OR practice AND guideline OR clinical AND protocols OR treatment AND protocol) AND TITLE-ABS-KEY (prisoners OR prisoner OR prisons OR penitentiaries OR penitentiary OR prison))
Medline via PubMed	(((((((((Guideline) OR (“Practice Guideline”)) OR (“Clinical Protocols”)) OR (“Protocol, Clinical”)) OR (“Treatment Protocols”)) OR (“Treatment Protocol”)) AND (Prisoners)) OR (Prison)) OR (Penitentiary)) AND (Hypertension) OR (“Diabetes Mellitus”)
Academic Search Premier — ASP (EBSCOhost)	(((((((((Guideline) OR (Practice Guideline)) OR (Clinical Protocols)) AND (((((((Prisoners) OR (Prisoner)) OR (Prison)) OR (Penitentiaries)) OR (Penitentiary) AND (Hypertension) OR (“Diabetes Mellitus”)
Web of Science	ALL=((((((((((Guideline) OR (“Practice Guideline”)) OR (“Clinical Protocols”)) OR (“Protocol, Clinical”)) OR (“Protocols, Clinical”)) OR (“Clinical Protocol”)) OR (“Treatment Protocols”)) OR (“Protocols, Treatment”)) OR (“Treatment Protocol”)) AND (((((((Prisoners) OR (Prisoner)) OR (Prison)) OR (Penitentiaries)) OR (Penitentiary)))
EMBASE	('guideline'/exp OR guideline OR 'practice guideline'/exp OR 'practice guideline' OR 'clinical practice guideline' OR 'clinical protocols' OR 'protocols, clinical' OR 'protocols, treatment') AND (prisoners OR hostages OR prisoner OR prisons OR penitentiaries)
Cochrane Library	Guideline in Title Abstract Keyword AND Prisoners in Title Abstract Keyword OR Prison in Title Abstract Keyword OR Penitentiary in Title Abstract Keyword AND “Hypertension” in Title Abstract Keyword OR “Diabetes Mellitus” in Title Abstract Keyword
**Gray literature**	
Open Grey	Prisoners Guideline AND Prisoners Guideline AND “Hypertension” Guideline AND “Diabetes Mellitus”
Brazilian Digital Library of Theses and Dissertations	Guideline AND Prisoners Protocolos AND Prisões Hipertensão e prisão Diabetes e prisão
National Council of Justice	Hipertensão e prisão Diabetes e prisão
National Institute for Health and Clinical Excellence	Guideline OR Practice OR Protocols OR Treatment OR Protocol OR Clinical Protocols AND Prisoners OR Prisoner OR Prisons OR Penitentiaries OR Penitentiary OR Prison AND Hypertension OR “Diabetes Mellitus”
Brazilian Ministry of Health	Hipertensão e prisão Diabetes e prisão Guideline AND Prisoners Guideline AND “Hypertension” Guideline AND “Diabetes Mellitus”
World Health Organization	Guideline OR Practice OR Protocols OR Treatment OR Protocol OR Clinical Protocols AND Prisoners OR Hostage OR Prisoner OR Prisons OR Penitentiaries OR Penitentiary OR Prison AND Hypertension OR Diabetes Mellitus
Pan American Health Organization	Guideline OR “Clinical Protocols” AND Prisoners Guideline AND Prisoners Guideline AND Hypertension Guideline AND “Diabetes Mellitus”
Canadian Medical Association	Guideline AND Prisoners Guideline AND Hypertension Guideline AND “Diabetes Mellitus”
The National Commission for the Incorporation of Technologies in the Unified Health System (CONITEC)	Guideline AND Prisoners Guideline AND Hypertension Guideline AND “Diabetes Mellitus”
The magazine of the National Commission on Correctional Health Care	Guideline AND Prisoners Guideline AND Hypertension Guideline AND “Diabetes Mellitus”
National Health and Medical Research Council	Guideline AND Prisoners Guideline AND Hypertension Guideline AND “Diabetes Mellitus”
Centers for Disease Control and Prevention	Guideline AND Hypertension Guideline AND Prisoners Guideline AND “Diabetes Mellitus”
GIN — Guidelines International Network (https://guidelines.ebmportal.com)	Guideline AND Hypertension Guideline AND Prisoners Guideline AND “Diabetes Mellitus”
